# Crystal structure of 9-butyl-3-(9-butyl-9*H*-carbazol-3-yl)-9*H*-carbazole

**DOI:** 10.1107/S1600536814025367

**Published:** 2014-11-21

**Authors:** K. Stalindurai, C. Ramalingan, B. Sridhar, S. Selvanayagam

**Affiliations:** a Department of Chemistry, Kalasalingam University, Krishnankoil 626 126, India; b Laboratory of X-ray Crystallography, Indian Institute of Chemical Technology, Hyderabad 500 067, India; c Department of Physics & International Research Centre, Kalasalingam University, Krishnankoil 626 126, India

**Keywords:** crystal structure, carbazole derivatives, C—H⋯π inter­actions

## Abstract

In the title carbazole derivative, C_32_H_32_N_2_, the mol­ecule resides on a crystallographic twofold axis, which runs through the central C—C bond. The carbazole ring system is almost planar, with a maximum deviation of 0.041 (1) Å for one of the ring-junction C atoms. The crystal packing is stabilized by C—H⋯π inter­actions only, which form a *C*(7) chain-like arrangement along [110] in the unit cell.

## Related literature   

For general background to carbazole derivatives and their applications, see: Giraud *et al.* (2014 ▶); Bandgar *et al.* (2012 ▶); Gu *et al.* (2014 ▶); Wang *et al.* (2011 ▶); Thiratmatrakul *et al.* (2014 ▶); Shi *et al.* (2012 ▶); Tavasli *et al.* (2012 ▶); Kim *et al.* (2011 ▶); Zhuang *et al.* (2012 ▶). For the preparation of the title compound, see: Ramalingan *et al.* (2010 ▶). 
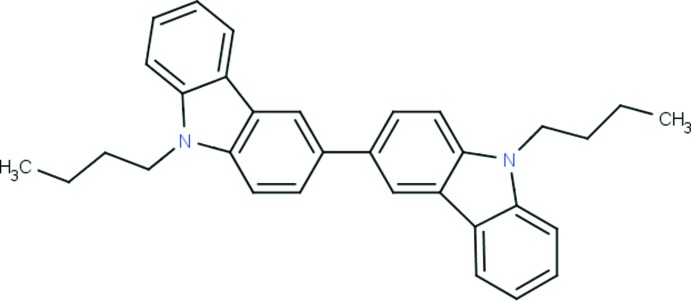


## Experimental   

### Crystal data  

C_32_H_32_N_2_
*M* *_r_* = 444.59
Monoclinic, 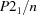

*a* = 5.6184 (4) Å
*b* = 11.0946 (7) Å
*c* = 19.4673 (13) Å
β = 95.982 (1)°
*V* = 1206.86 (14) Å^3^
*Z* = 2
Mo *K*α radiation
μ = 0.07 mm^−1^
*T* = 292 K
0.21 × 0.19 × 0.17 mm

### Data collection  

Bruker SMART APEX CCD area-detector diffractometer
13872 measured reflections
2928 independent reflections
2319 reflections with *I* > 2σ(*I*)
*R*_int_ = 0.026

### Refinement  

*R*[*F* ^2^ > 2σ(*F* ^2^)] = 0.061
*wR*(*F* ^2^) = 0.159
*S* = 1.12
2928 reflections
155 parameters
H-atom parameters constrained
Δρ_max_ = 0.24 e Å^−3^
Δρ_min_ = −0.19 e Å^−3^

### 

Data collection: *SMART* (Bruker, 2001 ▶); cell refinement: *SAINT* (Bruker, 2001 ▶); data reduction: *SAINT*; program(s) used to solve structure: *SHELXS97* (Sheldrick, 2008 ▶); program(s) used to refine structure: *SHELXL2013* (Sheldrick, 2008 ▶); molecular graphics: *ORTEP-3 for Windows* (Farrugia, 2012 ▶) and *PLATON* (Spek, 2009 ▶); software used to prepare material for publication: *SHELXL2013* and *PLATON*.

## Supplementary Material

Crystal structure: contains datablock(s) I, global. DOI: 10.1107/S1600536814025367/zq2229sup1.cif

Structure factors: contains datablock(s) I. DOI: 10.1107/S1600536814025367/zq2229Isup2.hkl

Supporting information file. DOI: 10.1107/S1600536814025367/zq2229Isup3.cml

**Fig. 1.**
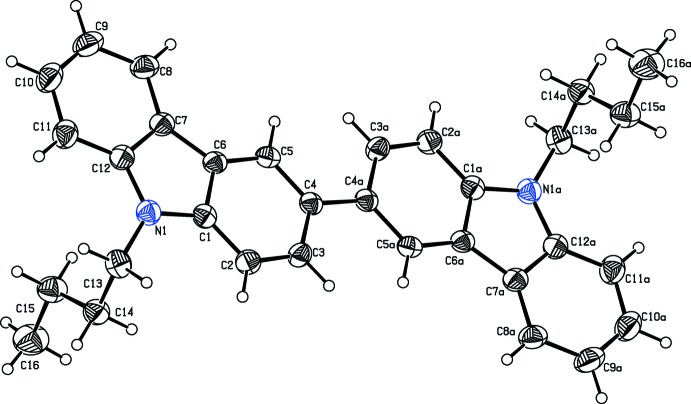
The molecular structure of the title compound, showing the atom-numbering scheme. Displacement ellipsoids are drawn at the 30% probability level.

. DOI: 10.1107/S1600536814025367/zq2229fig1.tif
The mol­ecular structure of the title compound, showing the atom-numbering scheme. Displacement ellipsoids are drawn at the 30% probability level.

**Fig. 2.**
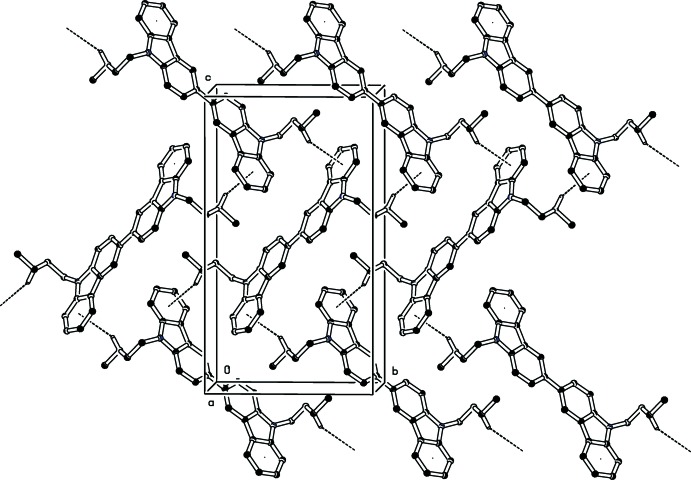
Molecular packing of the title compound, viewed along the a axis; C—H···π interactions are shown as dashed lines.For the sake of clarity, H atoms, not involved in hydrogen bonds, have been omitted for clarity.

. DOI: 10.1107/S1600536814025367/zq2229fig2.tif
Mol­ecular packing of the title compound, viewed along the a axis; C—H⋯π inter­actions are shown as dashed lines·For the sake of clarity, H atoms, not involved in hydrogen bonds, have been omitted for clarity.

CCDC reference: 1034987

Additional supporting information:  crystallographic information; 3D view; checkCIF report

## Figures and Tables

**Table 1:** Hydrogen-bond geometry (, ) *Cg* is the centroid of the C7C12 ring.

*D*H*A*	*D*H	H*A*	*D* *A*	*D*H*A*
C15H15*B* *Cg* ^i^	0.97	2.98	3.838(2)	148

Symmetry code: (i) 
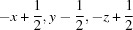
.

## supplementary crystallographic information

### S1. Comment

Carbazole based materials play vital roles in various areas of research. Various carbazole based heterocycles exhibit a diverse range of biological activities including pim kinase inhibitory (Giraud *et al.*, 2014), anti-inflammatory, antioxidant (Bandgar *et al.*, 2012), antimicrobial (Gu *et al.*, 2014), antitumor (Wang *et al.*, 2011), and anti-Alzheimer (Thiratmatrakul *et al.*, 2014) activities etc. On the other hand, this class of materials has been identified as potential ones for OLED applications (Shi *et al.*, 2012.; Tavasli *et al.*, 2012; Kim *et al.*, 2011; Zhuang *et al.*, 2012). As an intermediate for the development of new carbazole based materials for biological/OLED applications, a dibutylbicarbazole has been synthesized and single crystals were grown by slow evaporation in ethanol.

The X-ray study confirmed the molecular structure and atomic connectivity of the title compound, as illustrated in Fig. 1. The bond distance C4—C4^i^ of 1.488 (3) Å [symmetry code: (i) -x,-y+2,-z] confirms the single bond character. The sum of the angles at N1 (358.9°) is in accordance with sp^2^ hybridization.

The carbazole ring system is planar with a maximum deviation of -0.041 (1) Å for atom C7. The atom C13 attached to the carbazole ring system deviates by 0.250 (1) Å from the best plane of the carbazole ring system.

In addition to the van der Waals interactions, the molecular packing is influenced by intermolecular C—H···π interactions, such that atom H15B is 2.98 Å from the centroid of the phenyl ring (C7-C12) at (1/2-x, -1/2+y, 1/2-z) with a C15—H15B···.centroid angle of 148° and a C15···centroid distance of 3.838 (2) Å. This interactions form a C(7) chain like arrangement in the unit cell (Fig. 2).

### S2. Experimental

In a round-bottomed flask (250 ml), iron(III) chloride (44.80 mmol) in chloroform (100 ml) was taken under nitrogen atmosphere. Then, 9-butyl-9*H*-carbazole (11.20 mmol) (Ramalingan *et al.*, 2010) in chloroform (50 ml) was added in a drop-wise fashion and was stirred at ambient temperature for 1 hour. After the addition of a sodium hydroxide solution (10%), the organic phase was separated and the aqueous phase was extracted with chloroform. The combined organic phases were dried and concentrated to obtain the crude product which was dissolved in chloroform (15 ml) and reprecipitated slowly using methanol (200 ml). The product, thus, obtained was filtered, dried under vacuum at ambient temperature. Single crystals of (I) were obtained by slow evaporation of ethanol solution of the title compound at room temperature.

### S3. Refinement

H atoms were placed in idealized positions and allowed to ride on their parent atoms, with C—H distances of 0.93-0.97 Å, and *U*_iso_(H) = 1.5*U*_eq_(methyl C) and *U*_iso_(H) = 1.2*U*_eq_ for other C atoms.

### Crystal data

**Table:**

C_32_H_32_N_2_	*F*(000) = 476
*M**_r_* = 444.59	*D*_x_ = 1.223 Mg m^−^^3^
Monoclinic, *P*2_1_/*n*	Mo *K*α radiation, λ = 0.71073 Å
*a* = 5.6184 (4) Å	Cell parameters from 9858 reflections
*b* = 11.0946 (7) Å	θ = 2.4–27.2°
*c* = 19.4673 (13) Å	µ = 0.07 mm^−^^1^
β = 95.982 (1)°	*T* = 292 K
*V* = 1206.86 (14) Å^3^	Block, colourless
*Z* = 2	0.21 × 0.19 × 0.17 mm

### Data collection

**Table:**

Bruker SMART APEX CCD area-detector diffractometer	*R*_int_ = 0.026
Radiation source: fine-focus sealed tube	θ_max_ = 28.3°, θ_min_ = 2.1°
ω scans	*h* = −7→7
13872 measured reflections	*k* = −14→14
2928 independent reflections	*l* = −25→25
2319 reflections with *I* > 2σ(*I*)	

### Refinement

**Table:**

Refinement on *F*^2^	0 restraints
Least-squares matrix: full	Hydrogen site location: inferred from neighbouring sites
*R*[*F*^2^ > 2σ(*F*^2^)] = 0.061	H-atom parameters constrained
*wR*(*F*^2^) = 0.159	*w* = 1/[σ^2^(*F*_o_^2^) + (0.0782*P*)^2^ + 0.1445*P*] where *P* = (*F*_o_^2^ + 2*F*_c_^2^)/3
*S* = 1.12	(Δ/σ)_max_ < 0.001
2928 reflections	Δρ_max_ = 0.24 e Å^−^^3^
155 parameters	Δρ_min_ = −0.19 e Å^−^^3^

### Special details

**Table:**

Geometry. All esds (except the esd in the dihedral angle between two l.s. planes) are estimated using the full covariance matrix. The cell esds are taken into account individually in the estimation of esds in distances, angles and torsion angles; correlations between esds in cell parameters are only used when they are defined by crystal symmetry. An approximate (isotropic) treatment of cell esds is used for estimating esds involving l.s. planes.

### Fractional atomic coordinates and isotropic or equivalent isotropic displacement parameters (Å^2^)

**Table:**

	*x*	*y*	*z*	*U*_iso_*/*U*_eq_	
N1	0.3407 (2)	0.68730 (12)	0.15991 (7)	0.0515 (4)	
C1	0.2740 (3)	0.77442 (13)	0.11101 (8)	0.0453 (4)	
C2	0.3743 (3)	0.80464 (14)	0.05125 (9)	0.0520 (4)	
H2	0.5112	0.7659	0.0395	0.062*	
C3	0.2664 (3)	0.89298 (14)	0.00987 (8)	0.0498 (4)	
H3	0.3343	0.9134	−0.0301	0.060*	
C4	0.0581 (3)	0.95478 (12)	0.02450 (7)	0.0432 (3)	
C5	−0.0317 (3)	0.92638 (13)	0.08650 (8)	0.0464 (4)	
H5	−0.1644	0.9677	0.0991	0.056*	
C6	0.0733 (3)	0.83737 (14)	0.12995 (7)	0.0445 (4)	
C7	0.0185 (3)	0.78577 (14)	0.19467 (8)	0.0487 (4)	
C8	−0.1572 (3)	0.80724 (17)	0.23854 (9)	0.0601 (5)	
H8	−0.2688	0.8685	0.2289	0.072*	
C9	−0.1633 (4)	0.7361 (2)	0.29660 (9)	0.0711 (5)	
H9	−0.2801	0.7496	0.3263	0.085*	
C10	0.0034 (4)	0.64469 (19)	0.31106 (10)	0.0718 (6)	
H10	−0.0045	0.5980	0.3504	0.086*	
C11	0.1792 (4)	0.62115 (17)	0.26904 (9)	0.0627 (5)	
H11	0.2900	0.5598	0.2793	0.075*	
C12	0.1855 (3)	0.69250 (14)	0.21042 (8)	0.0502 (4)	
C13	0.5130 (3)	0.59184 (15)	0.15104 (9)	0.0572 (4)	
H13A	0.5533	0.5518	0.1950	0.069*	
H13B	0.6584	0.6275	0.1372	0.069*	
C14	0.4206 (3)	0.49911 (16)	0.09770 (9)	0.0580 (4)	
H14A	0.3929	0.5386	0.0531	0.070*	
H14B	0.5439	0.4388	0.0944	0.070*	
C15	0.1935 (3)	0.43619 (18)	0.11230 (10)	0.0661 (5)	
H15A	0.0679	0.4957	0.1143	0.079*	
H15B	0.2191	0.3973	0.1571	0.079*	
C16	0.1125 (4)	0.3428 (2)	0.05817 (12)	0.0911 (7)	
H16A	0.0976	0.3797	0.0133	0.137*	
H16B	−0.0396	0.3107	0.0674	0.137*	
H16C	0.2281	0.2789	0.0595	0.137*	

### Atomic displacement parameters (Å^2^)

**Table:**

	*U*^11^	*U*^22^	*U*^33^	*U*^12^	*U*^13^	*U*^23^
N1	0.0529 (7)	0.0499 (8)	0.0513 (8)	0.0010 (6)	0.0039 (6)	0.0007 (6)
C1	0.0430 (7)	0.0437 (8)	0.0487 (8)	−0.0055 (6)	0.0015 (6)	−0.0043 (6)
C2	0.0426 (8)	0.0532 (9)	0.0618 (10)	0.0013 (7)	0.0127 (7)	0.0005 (7)
C3	0.0476 (8)	0.0525 (9)	0.0511 (9)	−0.0050 (7)	0.0130 (7)	0.0015 (7)
C4	0.0439 (7)	0.0388 (7)	0.0468 (8)	−0.0065 (6)	0.0050 (6)	−0.0071 (6)
C5	0.0473 (8)	0.0457 (8)	0.0467 (8)	0.0007 (6)	0.0077 (6)	−0.0086 (6)
C6	0.0469 (8)	0.0437 (8)	0.0428 (8)	−0.0066 (6)	0.0049 (6)	−0.0089 (6)
C7	0.0543 (9)	0.0495 (8)	0.0420 (8)	−0.0086 (7)	0.0035 (6)	−0.0092 (7)
C8	0.0673 (11)	0.0648 (11)	0.0494 (9)	−0.0063 (8)	0.0124 (8)	−0.0130 (8)
C9	0.0836 (13)	0.0846 (14)	0.0484 (10)	−0.0165 (11)	0.0218 (9)	−0.0116 (9)
C10	0.0935 (14)	0.0744 (13)	0.0483 (10)	−0.0190 (11)	0.0109 (10)	0.0034 (9)
C11	0.0759 (12)	0.0602 (10)	0.0511 (10)	−0.0085 (9)	0.0019 (8)	0.0033 (8)
C12	0.0558 (9)	0.0498 (9)	0.0441 (8)	−0.0093 (7)	0.0015 (7)	−0.0056 (6)
C13	0.0491 (9)	0.0559 (10)	0.0651 (10)	0.0031 (7)	−0.0010 (7)	0.0033 (8)
C14	0.0574 (10)	0.0579 (10)	0.0594 (10)	0.0074 (8)	0.0090 (8)	0.0012 (8)
C15	0.0651 (11)	0.0693 (11)	0.0643 (11)	−0.0039 (9)	0.0089 (9)	−0.0126 (9)
C16	0.0988 (17)	0.0948 (16)	0.0795 (14)	−0.0229 (13)	0.0083 (12)	−0.0273 (13)

### Geometric parameters (Å, º)

**Table:**

N1—C1	1.381 (2)	C9—C10	1.389 (3)
N1—C12	1.382 (2)	C9—H9	0.9300
N1—C13	1.457 (2)	C10—C11	1.372 (3)
C1—C2	1.385 (2)	C10—H10	0.9300
C1—C6	1.408 (2)	C11—C12	1.392 (2)
C2—C3	1.369 (2)	C11—H11	0.9300
C2—H2	0.9300	C13—C14	1.515 (2)
C3—C4	1.411 (2)	C13—H13A	0.9700
C3—H3	0.9300	C13—H13B	0.9700
C4—C5	1.392 (2)	C14—C15	1.507 (3)
C4—C4^i^	1.488 (3)	C14—H14A	0.9700
C5—C6	1.391 (2)	C14—H14B	0.9700
C5—H5	0.9300	C15—C16	1.514 (3)
C6—C7	1.446 (2)	C15—H15A	0.9700
C7—C8	1.392 (2)	C15—H15B	0.9700
C7—C12	1.409 (2)	C16—H16A	0.9600
C8—C9	1.382 (3)	C16—H16B	0.9600
C8—H8	0.9300	C16—H16C	0.9600
			
C1—N1—C12	108.37 (13)	C9—C10—H10	119.0
C1—N1—C13	124.27 (14)	C10—C11—C12	117.53 (19)
C12—N1—C13	126.23 (14)	C10—C11—H11	121.2
N1—C1—C2	130.04 (14)	C12—C11—H11	121.2
N1—C1—C6	109.51 (13)	N1—C12—C11	129.16 (16)
C2—C1—C6	120.44 (14)	N1—C12—C7	109.34 (14)
C3—C2—C1	118.26 (14)	C11—C12—C7	121.50 (16)
C3—C2—H2	120.9	N1—C13—C14	112.98 (14)
C1—C2—H2	120.9	N1—C13—H13A	109.0
C2—C3—C4	123.69 (15)	C14—C13—H13A	109.0
C2—C3—H3	118.2	N1—C13—H13B	109.0
C4—C3—H3	118.2	C14—C13—H13B	109.0
C5—C4—C3	116.63 (14)	H13A—C13—H13B	107.8
C5—C4—C4^i^	122.27 (17)	C15—C14—C13	114.95 (15)
C3—C4—C4^i^	121.10 (17)	C15—C14—H14A	108.5
C6—C5—C4	121.26 (14)	C13—C14—H14A	108.5
C6—C5—H5	119.4	C15—C14—H14B	108.5
C4—C5—H5	119.4	C13—C14—H14B	108.5
C5—C6—C1	119.57 (13)	H14A—C14—H14B	107.5
C5—C6—C7	134.08 (14)	C14—C15—C16	112.63 (16)
C1—C6—C7	106.31 (14)	C14—C15—H15A	109.1
C8—C7—C12	119.42 (15)	C16—C15—H15A	109.1
C8—C7—C6	134.10 (16)	C14—C15—H15B	109.1
C12—C7—C6	106.44 (14)	C16—C15—H15B	109.1
C9—C8—C7	118.93 (18)	H15A—C15—H15B	107.8
C9—C8—H8	120.5	C15—C16—H16A	109.5
C7—C8—H8	120.5	C15—C16—H16B	109.5
C8—C9—C10	120.59 (18)	H16A—C16—H16B	109.5
C8—C9—H9	119.7	C15—C16—H16C	109.5
C10—C9—H9	119.7	H16A—C16—H16C	109.5
C11—C10—C9	122.02 (18)	H16B—C16—H16C	109.5
C11—C10—H10	119.0		
			
C12—N1—C1—C2	179.62 (16)	C1—C6—C7—C12	−1.41 (16)
C13—N1—C1—C2	−11.9 (2)	C12—C7—C8—C9	0.0 (2)
C12—N1—C1—C6	0.15 (16)	C6—C7—C8—C9	177.40 (16)
C13—N1—C1—C6	168.63 (13)	C7—C8—C9—C10	0.1 (3)
N1—C1—C2—C3	177.59 (14)	C8—C9—C10—C11	0.0 (3)
C6—C1—C2—C3	−3.0 (2)	C9—C10—C11—C12	−0.1 (3)
C1—C2—C3—C4	−0.2 (2)	C1—N1—C12—C11	178.61 (16)
C2—C3—C4—C5	3.2 (2)	C13—N1—C12—C11	10.4 (3)
C2—C3—C4—C4^i^	−176.60 (16)	C1—N1—C12—C7	−1.07 (17)
C3—C4—C5—C6	−2.9 (2)	C13—N1—C12—C7	−169.27 (14)
C4^i^—C4—C5—C6	176.86 (15)	C10—C11—C12—N1	−179.47 (16)
C4—C5—C6—C1	−0.1 (2)	C10—C11—C12—C7	0.2 (2)
C4—C5—C6—C7	−177.52 (15)	C8—C7—C12—N1	179.62 (14)
N1—C1—C6—C5	−177.26 (13)	C6—C7—C12—N1	1.54 (17)
C2—C1—C6—C5	3.2 (2)	C8—C7—C12—C11	−0.1 (2)
N1—C1—C6—C7	0.79 (16)	C6—C7—C12—C11	−178.17 (14)
C2—C1—C6—C7	−178.73 (14)	C1—N1—C13—C14	−69.5 (2)
C5—C6—C7—C8	−1.4 (3)	C12—N1—C13—C14	96.88 (19)
C1—C6—C7—C8	−179.08 (17)	N1—C13—C14—C15	−58.5 (2)
C5—C6—C7—C12	176.24 (15)	C13—C14—C15—C16	−178.80 (17)

Symmetry code: (i) −*x*, −*y*+2, −*z*.

### Hydrogen-bond geometry (Å, º)

Cg is the centroid of the C7–C12 ring.

**Table:**

*D*—H···*A*	*D*—H	H···*A*	*D*···*A*	*D*—H···*A*
C15—H15*B*···*Cg*^ii^	0.97	2.98	3.838 (2)	148

Symmetry code: (ii) −*x*+1/2, *y*−1/2, −*z*+1/2.
