# Supplementary figures and images for: Crystal structure of 9-butyl-3-(9-butyl-9H-carbazol-3-yl)-9H-carbazole
Source: Acta Crystallogr Sect E Struct Rep Online. 2014 Nov 21;70(Pt 12):o1283–4. doi: 10.1107/S1600536814025367 (PMC4257394; doi:10.1107/S1600536814025367)

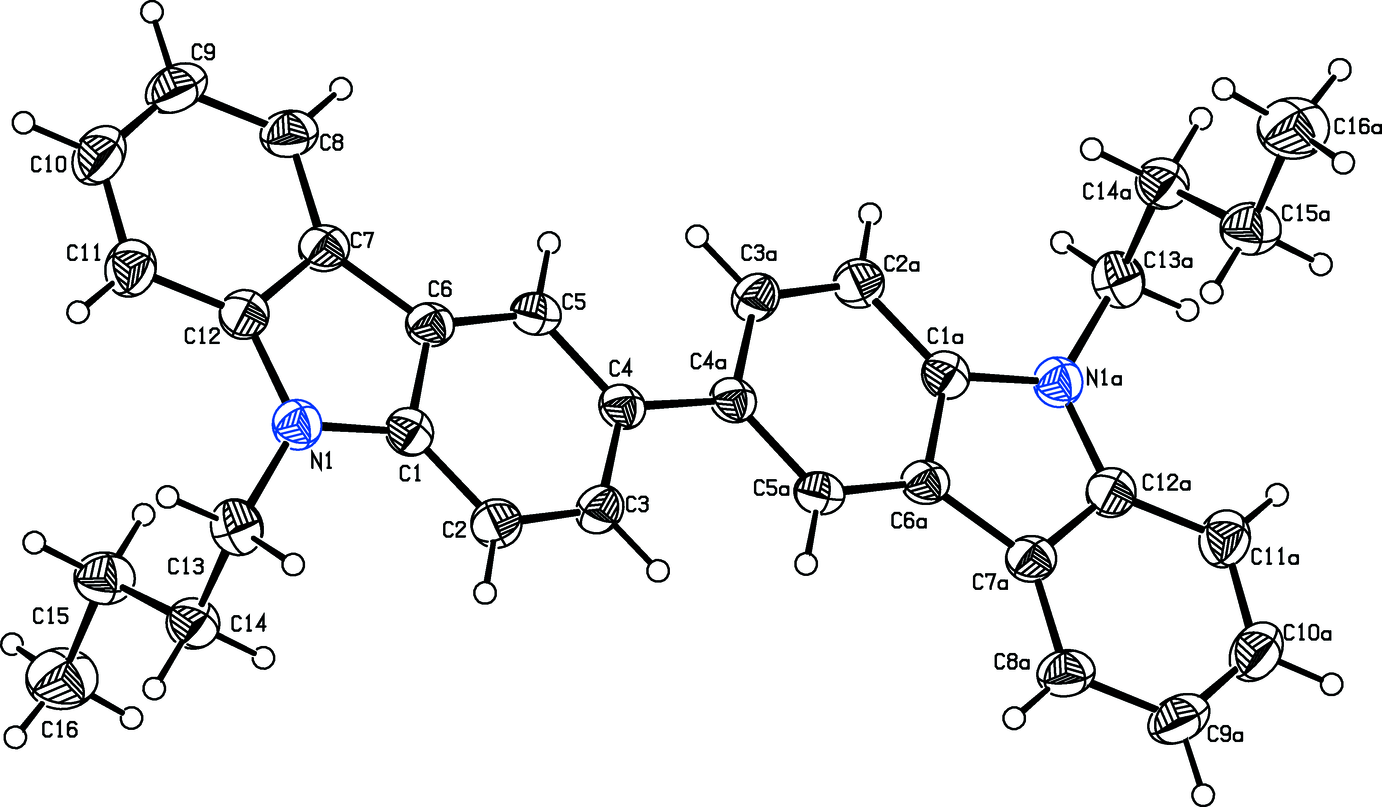

Supplement: Supplementary file 4 [file e-70-o1283-fig1.tif]

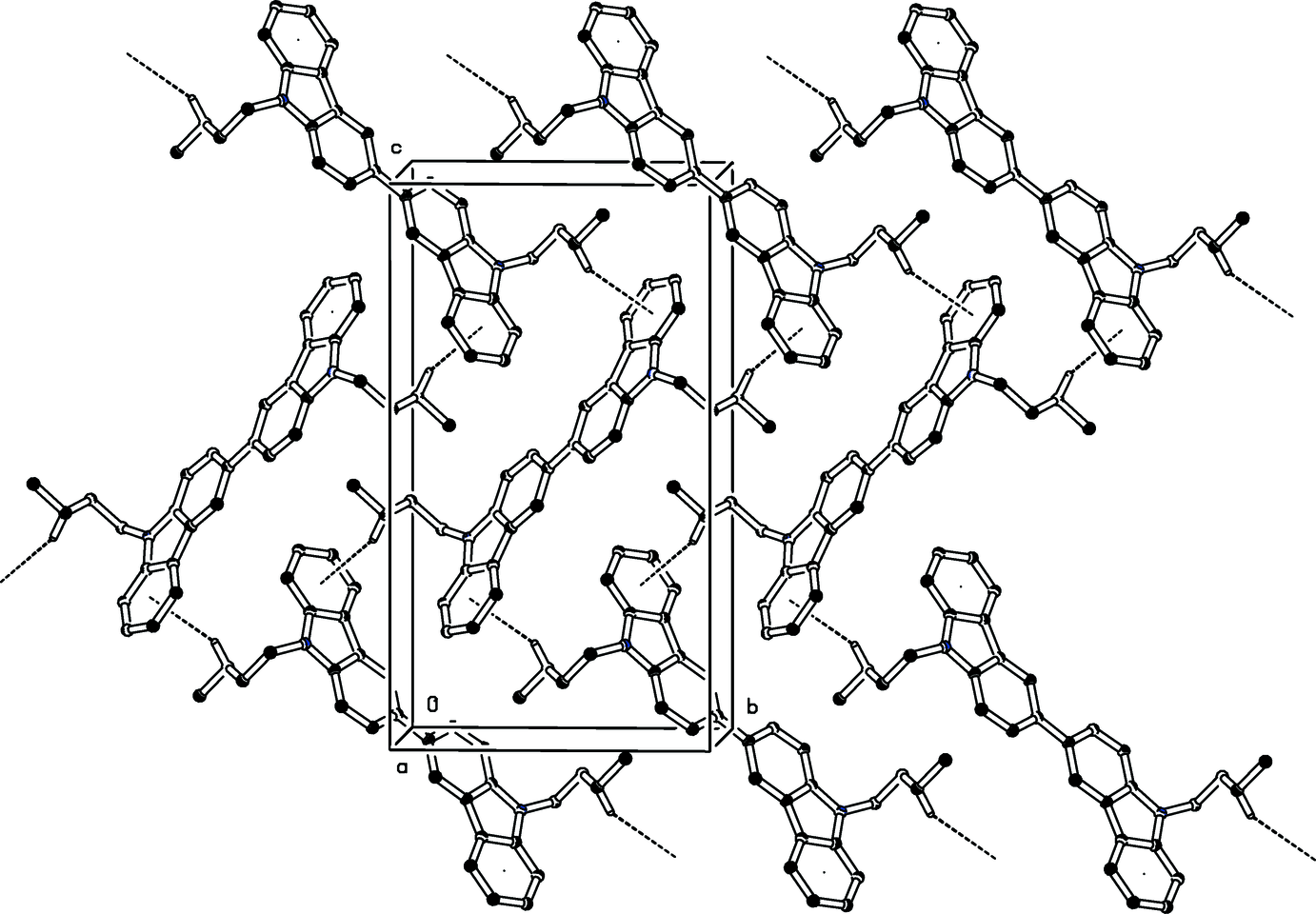

Supplement: Supplementary file 5 [file e-70-o1283-fig2.tif]
